# *In Vitro* Characterization of the eFlow Closed System Nebulizer with Glycopyrrolate Inhalation Solution

**DOI:** 10.1089/jamp.2017.1384

**Published:** 2018-06-01

**Authors:** Stephen Pham, Gary T. Ferguson, Edward Kerwin, Thomas Goodin, Alistair Wheeler, Andrea Bauer

**Affiliations:** ^1^ Sunovion Pharmaceuticals, Inc., Marlborough, Massachusetts.; ^2^ Pulmonary Research Institute of Southeast Michigan, Farmington Hills, Michigan.; ^3^ Clinical Research Institute of Southern Oregon, Inc., Medford, Oregon.

**Keywords:** COPD, eFlow Closed System, glycopyrrolate, nebulizers

## Abstract

***Background:*** Glycopyrrolate administered by a novel, investigational eFlow^®^ Closed System (CS) nebulizer (eFlow CS) is being evaluated for the maintenance treatment of chronic obstructive pulmonary disease (COPD). The eFlow CS is a hand-held, vibrating membrane nebulizer optimized to deliver 1 mL of glycopyrrolate solution into the lung in <3 minutes. Clinical studies have shown improvements in lung function of subjects treated with nebulized glycopyrrolate.

***Methods:*** The aerosol performance of the eFlow CS nebulizer was characterized by delivered dose, aerodynamic droplet size distribution and nebulization time. Simulated use nebulizer performance over 60 days was assessed by volume median diameter (VMD), nebulized amount, and nebulization time. Nebulization outputs were assayed to ensure adequate delivery of glycopyrrolate with an acceptable impurity profile. Aerosol condensates were analyzed for glycopyrrolate concentration and impurities by ultra-high-performance liquid chromatography and compared with non-nebulized samples.

***Results:*** The mean mass median aerodynamic diameter, geometric standard deviation, and fine particle fraction were 3.7 μm, 1.7, and 72%, respectively, and independent of formulation strength (25 and 50 μg/mL). Delivered dose was 88% of the nominal dose for both formulation strengths. The mean delivered dose, assessed by breathing simulation, was 56.8% for 25 μg/mL and 62.6% for 50 μg/mL. Nebulization times were 1–2.5 minutes with no apparent increasing trend with use over a 60-day period. The nebulized amount showed no significant changes, whereas the VMD showed a slight, but not pharmaceutically relevant, increase (0.1–0.2 μm) after 60-day simulated use. Glycopyrrolate concentration and impurity levels of nebulized samples were statistically similar to those of non-nebulized samples.

***Conclusion:*** The eFlow CS generates glycopyrrolate aerosols with high delivered dose, short treatment time, and small droplet size with narrow size distribution suitable for central and peripheral airway deposition. The unit dose vial mitigates medication misuse and ensures dose uniformity. Results support the use of glycopyrrolate/eFlow CS for the treatment of COPD.

## Introduction

Currently, there are no approved nebulized long-acting muscarinic antagonists (LAMAs) for the treatment of chronic obstructive pulmonary disease (COPD). Nebulized short-acting beta agonists (SABAs), short-acting muscarinic antagonists (SAMAs), SABA/SAMA combinations, and long-acting beta agonists used for COPD treatment are administered by standard jet nebulizers that are not drug product–device specific. The standard jet nebulizers are relatively low cost and easy to use, but have limited portability,^(1)^ many have poor delivery efficiency^(1–4)^ and long nebulization times,^(1,3,5)^ and their performances vary widely from one nebulizer to another.^(3,6)^

To address these unmet needs, Sunovion has developed a glycopyrrolate inhalation solution (SUN-101; Sunovion Pharmaceuticals, Inc., Marlborough, MA) for use in combination with the eFlow^®^ Closed System (eFlow CS) nebulizer. Glycopyrrolate is an established antimuscarinic adjunctive therapy and potent bronchodilator. It has been available as an injectable solution, Robinul^®^, for 30 years to reduce salivary, tracheobronchial, and pharyngeal secretions preoperatively; reduce the volume and free acidity of gastric secretions; and block cardiac vagal inhibitory reflexes during induction of anesthesia and intubation.

Recently, Seebri^®^ (glycopyrrolate, 15.6 μg twice daily [b.i.d.]), Utibron^®^ (indacaterol maleate/glycopyrrolate, 27.5 μg/15.6 μg b.i.d.) dry-powder inhalation (DPI) formulations, and Bevespi Aerosphere^®^ (glycopyrrolate/formoterol fumarate, 9.0 μg/4.8 μg per puff, delivered as 2 puffs b.i.d.) pressurized metered-dose inhaler (MDI) were approved in the United States for the maintenance treatment of COPD. SUN-101 (glycopyrrolate inhalation solution for nebulization) was developed for use in combination with the eFlow CS nebulizer by patients with moderate-to-very-severe COPD who cannot use MDI and DPI inhalers effectively (e.g., due to the inability to inhale quickly with force, coordinate single-breath actuation, or hold breath for the required time after dose delivery), as it allows the patient to take the dose using tidal breathing.

The eFlow CS nebulizer is a novel electronic nebulizer developed by PARI Pharma GmbH (Starnberg, Germany), modified from the eFlow^®^ technology platform of the Food and Drug Administration (FDA)-cleared electronic nebulizers used to administer medication for cystic fibrosis (e.g., Altera^®^, eRapid^®^). The eFlow CS nebulizer provides short nebulization times, silent operation, and portability for patients who require long-term, twice-daily therapy for disease management. More importantly, the eFlow CS nebulizer generates a gentle, soft aerosol mist of the drug solution with a droplet size distribution and acceptable respirable fraction suitable for central and peripheral lung deposition.^(7,8)^

Unlike open-system FDA-cleared devices based on the eFlow technology, the eFlow CS utilizes a unique, ready-to-use, unit dose, blow–fill–seal drug vial specifically designed to be placed into the medication cap of the device handset, which means that the patient does not have to open a drug vial and transfer the contents into the nebulizer.^(9)^ Subsequently, the drug vial is pierced by the device and the content is transferred into the handset without any patient manipulation of the drug product. This design is intended to prevent misuse with unintended medications and manual transfer of the contents into the nebulizer by the patient, thereby eliminating errors in dosing while improving dose uniformity.

**FIG. 1.**
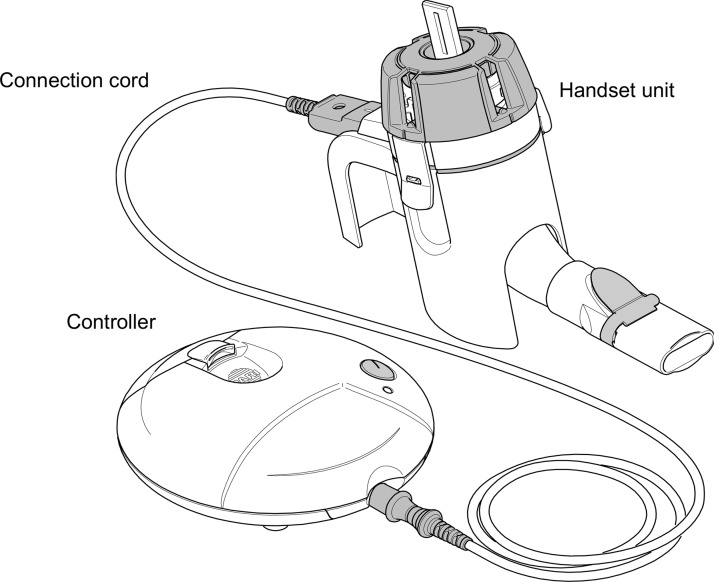
eFlow^®^ Closed System main components: handset unit, controller, and connection cord.

The glycopyrrolate/eFlow CS drug–device combination product was prospectively developed and optimized for aerosol droplet size for treating COPD and for usability by COPD patients. In the development of the glycopyrrolate/eFlow CS combination product, nine formative studies (*n* = 147, COPD patients and caregivers) and one final validation usability study (*n* = 91) were conducted, which resulted in iterative device optimizations to ensure that the target population is able to use the device safely and effectively. This culminated in the final design of the eFlow CS device, shown in Figure 1, used in the Glycopyrrolate for Obstructive Lung Disease via Electronic Nebulizer (GOLDEN) clinical development program.

Similar to the eFlow-based Altera nebulizer marketed for cystic fibrosis in which the handset is replaced every 28 days, the eFlow CS handset is intended to be replaced on a monthly basis. It has been reported that the vibrating membrane aerosol head unit has a limited lifespan when the device is not used in compliance with the instructions for use (IFU; i.e., lack of or no cleaning),^(10)^ which was also observed during the glycopyrrolate/eFlow CS Phase III clinical studies. While the eFlow CS can be used for much longer than 30 days when operated and maintained in accordance with the IFU (unpublished data), a 30-day replacement approach will be employed for glycopyrrolate inhalation solution to ensure optimal performance for patients.

This article summarizes the results of various *in vitro* studies conducted to characterize the performance of the glycopyrrolate/eFlow CS nebulizer drug–device combination that support the observed lung function improvements in subjects with moderate-to-very-severe COPD in the Phase II and III studies.

## Materials and Methods

### Aerosol performance characterization

The aerosol performance of the eFlow CS nebulizer, which included aerosol droplet size distribution and delivered dose, was determined by Next Generation Impactor (NGI) and by a breathing simulator, respectively. Thirty investigational eFlow CS nebulizers selected from seven production lots were used in the study, and 15 devices were tested with both glycopyrrolate dosing regimens (1 mL of the 25 or 50 μg/mL solution).

The glycopyrrolate test samples were obtained from three clinical lots (25 μg/mL lot numbers: PC153, RI177, RI178; 50 μg/mL lot numbers: RH234, RI123, RL093) manufactured by Holopack Verpackungstechnik GmbH (Sulzbach-Laufen, Germany). A set of five dedicated eFlow CS devices were used for each glycopyrrolate drug product lot. Each device was tested in single measurement for NGI and duplicate measurements for delivered dose.

### Simulated use testing

A 60-day simulated use study was conducted to assess and qualify the performance of the eFlow CS device at twice the proposed clinical use by patients.

Six eFlow CS devices were used in accordance with the Phase III clinical trial IFU to simulate 60 days, twice-daily use, and glycopyrrolate 25 μg/mL was used as the test solution. A total of 120 nebulization cycles were tested on each device (2 doses per day × 60 days of use).

Nebulizer cleaning was performed after each use as per the IFU, and the aerosol head was backwashed using the *easycare* cleaning accessory (operating the aerosol head in the reverse direction to clear the membrane micro-orifices) with 1 mL of water once per week. The handset, including the aerosol head, was disinfected (optional for Phase III clinical studies) with Control III^®^ after every 14 uses (equivalent to weekly use). Nebulization time, defined as the time taken for the nebulizer to generate aerosol mist, was measured for every use. Nebulizer performance was assessed by volume median diameter (VMD) by laser diffraction, total output rate, and nebulized amount by gravimetric analysis, before and after the 60-day simulation test.

### Impurity characterization of nebulized glycopyrrolate

The impurity profile of glycopyrrolate aerosols was assessed using two investigational eFlow CS devices. Glycopyrrolate drug vials were randomly selected from the 25 and 50 μg/mL clinical batch for testing.

A 20-mL Type I glass vial was fitted into the nebulizer outlet. The nebulizer was tilted slightly downward so that the aerosol output and its condensate were collected into the glass vial. Multiple drug vials were nebulized and the aerosol condensate was pooled to obtain ∼1 to 2 mL in the glass vial, the amount needed for the drug assay and impurities analysis. Three test samples were collected in separate, clean glass vials.

All samples were analyzed using a stability-indicating ultra-high-performance liquid chromatography (UHPLC) method, which used gradient reversed-phase elution with ultraviolet (UV) detection at 220 nm. The method is validated for specificity, accuracy, precision, and robustness. The range of the method is 0.0125–0.75 μg/mL and the level of sensitivity is 0.05% of a 25 μg/mL dose.

### Aerodynamic droplet size distribution by NGI

The aerodynamic droplet size distributions of glycopyrrolate 25 and 50 μg/mL aerosols were determined by NGI in accordance with U.S. Pharmacopoeia (USP) <1601>. The NGI body and components, including the cups, micro-orifice collection filter, and induction port, were cooled at 5°C for at least 90 minutes before collection. NGI testing was performed in a controlled environment of 23 ± 2°C and 50 ± 5% relative humidity (RH) within 5 minutes of NGI removal from the cold condition. The NGI was operated at an airflow rate of 15.0 ± 0.75 L/min. Aerosol was collected until at least 10 seconds after the nebulizer automatically shut off, indicated by an audio indicator (i.e., buzzer) and visual light-emitting diode (LED) feedback. The nebulizer and the NGI samples were recovered as per validated procedures and the drug contents were analyzed by HPLC.

Mass median aerodynamic diameter (MMAD), geometric standard deviation (GSD), fine particle dose (FPD, amount below 5 μm), and fine particle fraction (FPF, % below 5 μm) were determined using validated Copley Inhaler Testing Data Analysis Software (Copley Scientific, Ltd., UK). Aerosol generation time (time from start of nebulization to end of aerosol mist generation) of each nebulization was visually measured. Given the number of devices tested for each strength (*n* = 15 devices/strength) and that the NGI test method has been validated for its intended use, aerosol droplet size distribution measurement by NGI was performed in a single measurement for each eFlow CS device.

### Delivered dose by continuous flow

The delivered dose of nebulized glycopyrrolate by continuous flow was conducted for quality control purposes using the following validated procedure. A dose unit sampling apparatus (DUSA) was used for aerosol collection. The DUSA tube was prepared by placing a 47 mm metal screen and two 47 mm Whatman 934-AH glass filters on the filter holder in the DUSA tube and screwed in place. To prevent oversaturation of the Whatman micro fiberglass filter, three Kimwipe sheets were placed in the DUSA tube in a random manner. One end of the DUSA tube was connected to an eFlow CS nebulizer through a mouthpiece adapter and the other end of the DUSA tube was attached to a vacuum source with a constant airflow rate of 15 ± 1 L/min through the eFlow CS nebulizer and the DUSA tube.

The nebulization was started and aerosol collected until the eFlow CS nebulizer turned off, as indicated by the audio buzzer and the LED light. The drug contents collected in the DUSA tube and the residual drug in the nebulizer were then extracted as per the validated procedure and analyzed by HPLC. Each device was tested in duplicate.

### Delivered dose by breathing simulation

The delivered dose of glycopyrrolate by breathing simulation (for dose-labeling purposes) was conducted as per USP <1601> with adult breathing pattern (500 mL tidal volume, 15 breaths per minute, and inhalation:exhalation ratio of 1:1, sinusoidal waveform). Before aerosol collection, the breathing simulation apparatus (Copley breathing simulator model BRS1000; Copley Scientific, Ltd.) was run for 1 minute to allow for equilibration. The eFlow CS nebulizer was then attached to a Respirgard II™ filter (Vital Signs, Inc., NJ) and connected to the breathing simulation apparatus. The breathing simulator and the eFlow CS nebulizer were simultaneously started, and aerosol was collected until the eFlow CS nebulizer turned off, as indicated by the audio indicator and LED visual signal.

The filter and nebulizer were disassembled, the drug on the filter and in the nebulizer was extracted, and the drug concentrations were determined by HPLC. Each device was tested in duplicate.

### VMD by laser diffraction

VMDs of aerosols generated by the eFlow CS before and after the 60-day simulated use study were determined using a Malvern Mastersizer X Particle Size Analyzer (Malvern Instruments, MA). An eFlow CS ampoule filled with 1 mL of saline solution was nebulized in a temperature/RH controlled chamber of 23 ± 2°C/50 ± 5% RH attached to the Malvern Mastersizer. Aerosol was entrained across the laser beam of the particle size analyzer at 20 L/min. The eFlow CS nebulizer was operated for 70 seconds and the measurement started 10 seconds after the controller was activated. An average droplet size distribution was calculated based on five 5-second measurements with an interval of 10 seconds.

### Nebulized amount

A nebulizer handset installed with an eFlow CS ampoule filled with 1 mL of saline solution was accurately weighed by a calibrated laboratory balance XS603S DR (Mettler Toledo, OH). The assembled handset was then placed in a test chamber with controlled temperature and RH (23 ± 2°C/50 ± 5% RH). While air was entraining through the measurement chamber at 20 L/min, the nebulizer was switched on and allowed to nebulize until it automatically shut off. The nebulizer handset was then reweighed and the nebulized amount was determined gravimetrically by taking the difference in the nebulizer handset weight before and after nebulization.

### HPLC method

The HPLC method for determination of the aerodynamic droplet size distribution and the delivered dose of glycopyrrolate (i.e., glycopyrrolate content) of the nebulized solutions used an isocratic reversed-phase elution and UV detection at 220 nm. The method has been validated for specificity, accuracy, and precision. The range of the method is 0.7% (0.007 μg/mL) to 500% (5.0 μg/mL) of the nominal method concentration of glycopyrrolate 1 μg/mL when determining aerodynamic droplet size distributions, and 0.7% (0.007 μg/mL) to 80% (0.8 μg/mL) of the nominal method concentration when quantifying the delivered dose.

## Results

### Aerosol performance

The MMAD, GSD, FPD, and FPF are summarized in Table 1. Both glycopyrrolate product strengths (25 and 50 μg/mL) have similar MMAD, GSD, and FPF. The FPD was found to be directly proportional to dose strength. The average nebulization time was 2 minutes.

**Table 1.** Aerosol Performance Characteristics of eFlow Closed System Nebulizers with Glycopyrrolate (*N* = 15, Mean ± Standard Deviation)

*Glycopyrrolate strength*	*Nebulization time (minutes)*	*MMAD (μm)*	*GSD*	*FPD (μg)*	*FPF (%)*	*NGI recovery (% nominal dose)*
25 μg/mL	1.88 ± 0.23	3.71 ± 0.26	1.66 ± 0.06	15.20 ± 1.26	72.28 ± 5.57	95.61 ± 1.90
50 μg/mL	1.92 ± 0.14	3.74 ± 0.20	1.65 ± 0.04	30.44 ± 1.51	71.65 ± 4.34	94.94 ± 1.75

CS, Closed System; FPD, fine particle dose; FPF, fine particle fraction; GSD, geometric standard deviation; MMAD, mass median aerodynamic diameter; NGI, Next Generation Impactor.

**FIG. 2.**
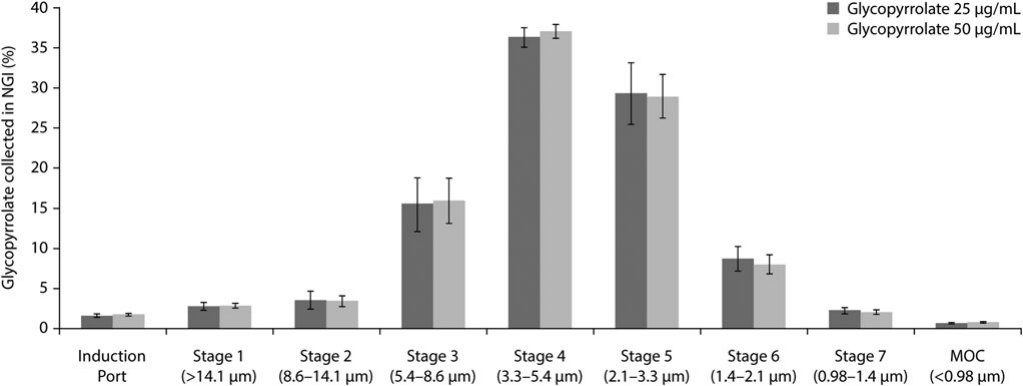
NGI stage-by-stage deposition profile of glycopyrrolate aerosols with effective size cutoff (*n* = 15, mean ± SD). MOC, micro-orifice collection; NGI, Next Generation Impactor; SD, standard deviation.

Figure 2 shows NGI stage-by-stage deposition profiles of the 25 and 50 μg/mL glycopyrrolate aerosols, normalized to the amount of drug collected ex-device. The NGI deposition profiles of aerosols generated from the 25 and 50 μg/mL glycopyrrolate strengths are similar, indicating that the aerodynamic size distribution of glycopyrrolate aerosol is independent of formulation strength.

Delivered-dose data determined by continuous flow and breathing simulation methods are summarized in Table 2. The mean delivered dose (% nominal dose) of glycopyrrolate by continuous flow method was similar for both formulation strengths (25 μg/mL, 87.7%; 50 μg/mL, 88.2%), whereas the mean delivered dose (% nominal dose) by breathing simulation showed a slight difference between the two strengths (25 μg/mL, 56.8%; 50 μg/mL, 62.6%). The delivered dose and the total recovery determined by breathing simulation method are less than those determined by the continuous flow method of the same strength. In general, percent of dose delivered by customized eFlow technology-based nebulizers is considerably greater than the percent of dose delivered reported for standard jet nebulizers.^(4,7)^

**Table 2.** Delivered Dose by Continuous Flow and by Breathing Simulation of Glycopyrrolate

	*Results (n* = *30, mean ± SD)*				
*Glycopyrrolate strength*	*Nebulization time (minutes)*	*Delivered dose (μg)*	*Delivered dose (% nominal dose)*	*Residual dose (% nominal dose)*	*Total recovery (% nominal dose)*
Delivered dose by continuous flow method					
25 μg/mL	1.90 ± 0.29	21.93 ± 1.16	87.72 ± 4.65	13.19 ± 4.23	100.94 ± 2.70
50 μg/mL	1.99 ± 0.21	44.11 ± 1.43	88.23 ± 2.86	9.21 ± 3.02	99.55 ± 1.92
Delivered dose by breathing simulation method					
25 μg/mL	2.02 ± 0.31	14.20 ± 1.21	56.80 ± 4.83	25.55 ± 5.85	85.36 ± 2.13
50 μg/mL	2.06 ± 0.24	31.29 ± 1.87	62.57 ± 3.74	25.56 ± 4.22	88.14 ± 1.56

SD, standard deviation.

### Simulated use testing

**FIG. 3.**
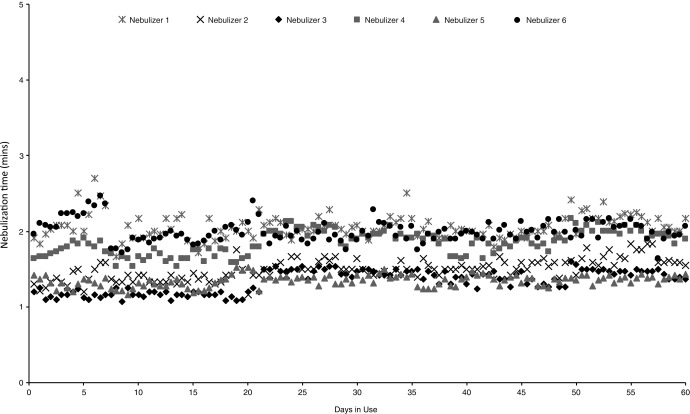
Nebulization times from 60-day simulated use study.

The nebulization times of all six eFlow CS devices showed no appreciable changes throughout the 60-day simulated use study (Fig. 3), and there were no changes in the visual appearance of the aerosol heads before and after the study.

Table 3 presents the VMD and nebulized amount measured before and after completion of the 60-day simulated use study. Sample pair-wise *t*-test of the VMD showed that, although small, there is a statistical increase in the VMD after completion of the 60-day simulation study (*p* < 0.05). The nebulized amount showed no statistical change before and after the study (*p* > 0.05).

**Table 3.** Device Performance Characteristics Before and After 60-Day Simulated Use Study

	*VMD (μm)*		*Nebulized amount (g)*	
*eFlow CS nebulizer*	*Before simulated use study*	*Postsimulated use study*	*Before simulated use study*	*Postsimulated use study*
Nebulizer 1	3.0	3.1	0.95	0.92
Nebulizer 2	3.6	3.8	0.89	0.92
Nebulizer 3	3.5	3.6	0.94	0.96
Nebulizer 4	3.2	3.4	0.93	0.92
Nebulizer 5	3.4	3.6	0.97	0.96
Nebulizer 6	3.1	3.3	0.92	0.94
Mean ± SD	3.3 ± 0.2	3.5 ± 0.3	0.93 ± 0.03	0.94 ± 0.02

CS, closed system; VMD, volume median diameter; SD, standard deviation.

### Impurity characterization of nebulized glycopyrrolate

The results of the assay and impurity profile of the drug product before and after nebulization are summarized in Table 4. Impurities are reported if present at ≥0.10%. The assay and impurity results for the prenebulized and nebulized glycopyrrolate samples are similar for both sets of eFlow CS nebulizers tested. There were no noticeable differences in the glycopyrrolate concentration or the impurity levels (knowns and unknowns), and no new impurities were reported. These results demonstrate that the eFlow nebulization process does not change the drug concentration and impurity profile of glycopyrrolate.

**Table 4.** Assay and Related Substances Results for Glycopyrrolate Pre- and Postnebulization Test Samples

			*Postnebulized (*n = *3, mean ± SD)*	
*Glycopyrrolate strength*	*Drug assay/related substances*	*Prenebulized*	*Nebulizer 1*	*Nebulizer 2*
25 μg/mL	Active (% nominal concentration)	98.70	98.64 ± 0.32	98.69 ± 0.15
	Known impurity 1 (%)	0.15	0.15 ± 0.01	0.14 ± 0.00
	Known impurity 2 (%)	0.28	0.29 ± 0.01	0.28 ± 0.00
	Largest unknown impurity (%)	0.36	0.33 ± 0.02	0.34 ± 0.02
	Total impurities (%)	0.78	0.77 ± 0.02	0.76 ± 0.01
50 μg/mL	Active (% nominal concentration)	98.70	98.31 ± 0.33	98.76 ± 0.78
	Known impurity 1 (%)	<0.10	<0.10	<0.10
	Known impurity 2 (%)	0.23	0.24 ± 0.00	0.24 ± 0.00
	Largest unknown impurity (%)	0.14	0.14 ± 0.01	0.14 ± 0.01
	Total impurities (%)	0.38	0.38 ± 0.01	0.38 ± 0.01

SD, standard deviation.

## Discussion

The performance characteristics of the eFlow CS vibrating membrane nebulizer support its use for the administration of glycopyrrolate drug solution as a potential maintenance treatment for COPD. The specifically engineered eFlow vibrating membrane technology, and the uniquely designed CS ampoule of the eFlow CS nebulizer, produces glycopyrrolate aerosols with optimized acceptable respirable fractions and dose uniformity under routine laboratory and simulated stressed-use conditions.

eFlow technology has also been used to deliver other classes of inhaled pulmonary therapies, including: Tolero^®^ for Vantobra^®^ (tobramycin) as approved in the European Union; Zirela^®^ for Quinsair^®^ (levofloxacin) as approved in the European Union and Canada; Altera for Cayston^®^ (aztreonam) as approved in the United States, the European Union, Canada, Australia, and Israel; and eFlow *rapid* for ColiFin^®^ (colistimethate sodium) as approved in Germany, Austria, Italy, Spain, the United Kingdom, and the Netherlands.

When tested at constant airflow rate of 15 L/min, 88% of the nominal dose was collected as aerosol with good reproducibility (% relative standard deviation ≤5%). The difference in delivered dose between the constant flow and breathing simulation methods is expected because of aerosol loss to the environment during the exhalation phase associated with the breathing simulation method.

The eFlow CS device, when used with glycopyrrolate in accordance with the IFU, can deliver the 1 mL dose in about 1–2.5 minutes with no apparent change in nebulization time through 120 doses, equivalent to 60 days of twice-a-day use. Aerosol characteristics tested with saline solution before and after the simulation study showed a small, but not pharmaceutically relevant, increase in the VMD (0.1–0.2 μm) and no statistical change in the nebulized amount. The nebulization times, aerosol, and device performance characteristics do not change appreciably over 60 days of simulated use. While the *easycare* was used in the study for backwashing the eFlow CS aerosol head every week, this accessory will not be used in the commercial setting because the handset will be replaced every 30 days, obviating the need for weekly supplemental backwashing.

The assay and impurity results of glycopyrrolate samples tested before nebulization and as aerosol condensate are similar. Unlike jet nebulizers, where the drug concentration changes appreciably during the course of nebulization due to evaporation and recirculation, the glycopyrrolate assay values do not change from nebulization. The absence of new impurities or increase in known and unknown impurities from nebulization demonstrated that glycopyrrolate is compatible for use with the eFlow CS nebulizer and supports its use in the clinic.

## Conclusions

The eFlow CS nebulizer is a novel portable device capable of nebulizing a glycopyrrolate drug solution, with high delivery efficiency, short treatment time, and producing aerosols that are suitable for central and peripheral airway deposition. The nebulization process, utilizing the well-proven vibrating membrane technology, has no adverse impact on glycopyrrolate, the active ingredient, and its related impurities that would otherwise present a medical risk to patients. The unique closed-system vial design produced a highly uniform delivered dose, in addition to eliminating human errors from using unintended medication or improper dosing.

The use of the eFlow CS device with glycopyrrolate in Phase II and III clinical studies demonstrated statistically significant and clinically important improvements in lung function with acceptable safety profiles in subjects with moderate-to-very-severe COPD treated for up to 48 weeks.^(11–13)^ Therefore, the glycopyrrolate/eFlow CS drug–device combination product addresses an unmet medical need for a nebulized LAMA and represents a potential maintenance treatment for COPD.
